# Hypericum perforatum as a cognitive enhancer in rodents: A meta-analysis

**DOI:** 10.1038/srep35700

**Published:** 2016-10-20

**Authors:** Daniel Ben-Eliezer, Eldad Yechiam

**Affiliations:** 1 Industrial Engineering and Management, Technion City Haifa, Israel 3200001 Israel.

## Abstract

Considered an antidepressant and anti-anxiety agent, *Hypericum perforatum* affects multiple neurotransmitters in a non-competitive synergistic manner, and may have nootropic potential. We quantitatively reviewed the pre-clinical literature to examine if there is a cognitive-enhancing effect of *H. perforatum* in healthy rodents. Additionally, within these studies, we compared the effects observed in intact rodents versus those whose performance has been impaired, mostly through stress manipulations. The meta-analysis incorporated studies that examined the effect of *H. perforatum* versus placebo on memory indices of task performance. All analyses were based on weighting different studies according to their inverse variance. Thirteen independent studies (published 2000–2014) involving 20 experimental comparisons met our inclusion criteria. The results showed a large positive effect of *H. perforatum* on cognitive performance for intact, healthy rodents (*d* = 1.11), though a larger effect emerged for stress-impaired rodents (*d* = 3.10 for restraint stress). The positive effect on intact rodents was observed in tasks assessing reference memory as well as working memory, and was not moderated by the type of memory or motivation (appetitive versus aversive). Thus, while primarily considered as a medication for depression, *H. perforatum* shows considerable nootropic potential in rodents.

*Hypericum perforatum (H. perforatum)*, also known as St John’s wort, has been regarded as a medicinal herb for at least two thousand years, being first mentioned in the first century by the Greek herbalist Pedanios Dioskourides^1^. Traditionally, *H. perforatum* had been utilized for a variety of medical purposes such as the treatment of burns, wounds, hematomas, inflammations, and muscle pain^1^,^2^. It has also been used to reduce fearfulness and melancholy, and is depicted in various religious texts as a “demon chaser”^2^. This latter psychological effect has recently been rediscovered, with *H. perforatum* being found to reduce anxiety and depression^3^,^4^. Later research showed that it was as effective for reducing symptoms of mild to moderate depression as classic antidepressants such as tricyclic antidepressants (TCA)^5^ and selective serotonin reuptake inhibitors (SSRIs)^6^. Today, *H. perforatum* is an established medicine for mild to moderate depression, registered in many European countries^7^, and considered to have fewer side effects compared to other antidepressants^7^,^8^. In addition to its effect on depression and anxiety, it has been suggested in preclinical studies that *H. perforatum* also improves some aspects of cognitive functioning, particularly the acquisition and consolidation of memories^9^,^10^,^11^. However, this effect has not always been found to replicate in rodents^12^,^13^, while no benefit has been found in humans^14^,^15^. The main goals of the current paper are to clarify whether there is a cognitive-enhancing (nootropic) effect of *H. perforatum* in rodents, and to examine the relationship between the antidepressant and nootropic effects of *H. perforatum* by evaluating its effects in intact rodents versus those subjected to stress manipulations leading to impaired cognitive performance. We address these research questions by quantitatively reviewing the relevant pre-clinical studies that have examined intact animals. When these studies also examined performance-impaired animals (in a two by two design: impaired/intact × placebo/medication), we assessed the relative benefit of *H. perforatum* for intact rodents. In particular, we compared the effect for intact animals and those whose performance was impaired, while controlling for the design of the study, the dosage, and the experimental task.

*H. perforatum* includes at least seven different active ingredients^16^, among them hypericin and hyperforin are considered the primary constituents^7^,^17^. It has been demonstrated that these components have a unique combined pharmacological effect, inhibiting the reuptake of several neurotransmitters in a non-competitive synergistic manner^18^. Similar to other antidepressants, *H. perforatum* inhibits the reuptake of monoamine neurotransmitters (5-HT, noradrenaline, and dopamine)^18^,^19^,^20^, which increases the concentration of serotonin and other monoamines in the extracellular space in brain regions such as the hippocampus, thalamus, amygdala, and the prefrontal cortex^21^. Upregulation of 5-HT in these areas reduces negative affect from impinging into memory processes^22^,^23^, while upregulation of dopamine reduces background firing rate of neurons, thus decreasing non-task related activity and improving signal to noise ratio^24^. These monoaminergic effects are considered quite weak^8^,^16^,^18^; however, they are relatively broad in that not only the expression of 5-HT_1A_ receptors is upregulated as in most SSRIs, but also of 5-HT_2_ receptors^17^,^18^. Another effect of *H. perforatum* is blocking the binding of GABA^3^, which results in decreased central nervous system inhibition^25^. This arousal-related effect is considered to improve the consolidation of memory in the long term storage^26^. *H. perforatum* further regulates NMDA receptors^18^,^27^ which play a pivotal role in memory and nootropics^28^. The existence of these neuropharmacological pathways suggests that *H. perforatum* can enhance the cognitive performance of healthy intact animals, either because of its anti-anxiolytic effects which may alleviate task stress or through its effects on memory and task-related attention.

## Methods

A Google Scholar structured search using different keywords was conducted in order to find relevant studies (last search run on March 2016). No limits were applied for language and foreign papers were translated. We used the following search terms: “hypericum”, “animal”, and one of the following: “nootropic”, “cognitive enhancing”, “cognitive enhancers”, “memory enhancing”, or “memory enhancement”. We also included all relevant studies referred to by a previous comprehensive review^7^.

The following eligibility criteria were used: with respect to *study type*, we included only studies that examined the effect of *H. perforatum* versus placebo. No language, publication date, or publication status restrictions were imposed. With respect to *types of participants*, we included only animal studies with healthy intact animals. Thus, we included studies fulfilling the following two criteria: random assignment of animals to groups, and a complete design with at least one healthy, intact, control group treated with placebo and a healthy intact rodent group treated with *H. perforatum*. With respect to the *type of intervention*, we included any dosage of *H. perforatum* administered for any duration, versus placebo but not versus no medication at all. With respect to *types of outcome measure*, our analysis focused on learning and memory indices of task-performance. Further to this, we focused on response time measures of these indices (which were commonly reported). Studies or conditions involving tasks that focused on anxiolytic effects of *H. perforatum*, such as time to escape an unknown dangerous place, were excluded (because this examination confounds nootropic and antidepressant/anxiolytic effects). Methods of the analysis and inclusion criteria were specified in advance and documented. Eligibility assessment was performed independently in an unblinded standardized manner by two reviewers. Disagreements between reviewers were resolved by consensus (though no such case emerged).

We used a data extraction protocol based on the Cochrane Consumers and Communication Review Group’s data extraction template. One review author extracted the data from included studies and the second author checked the extracted data. When a paper lacked statistical data (averages, standard errors), we requested data from the authors. In cases of no response, we extracted means and SDs from figures using the ImageJ^®^ software (V. 1.48), following the protocol used in other reviews^29^,^30^.

Information was extracted from each included trial on: (1) characteristics of organisms (species, age), and the trial’s inclusion and exclusion criteria; (2) type of intervention (including type, dose, duration, and frequency of *H. perforatum* administration; versus placebo; and any impairment condition, and (3) type of outcome measure (task name and all indices of task performance). The primary measure was speed of performance. The tasks used in the different studies are shown in Table 1.

To examine the effect on different types of memory, we categorized the tasks into those assessing primarily working memory, reference memory (i.e., long term storage of fixed trial properties), and recognition memory. To ascertain whether the effect only emerged for avoidance-related responses we further categorized each task as appetitive or aversive and tested whether effect size may differ under these two categories. This categorization of tasks into memory types and appetitive/aversive motivation appears on Supplementary Table S1. Finally, each paper was reviewed to determine whether animals had access to food and water and were kept in ample temperature and humidity prior to the experiment, and the blinding of data collectors and outcome assessors.

The meta-analysis was based on all relevant tasks within the included papers. In cases where multiple dosages were used in different conditions, we considered the maximal one. However, in order to examine the moderating effect of dosage we conducted a secondary analysis including all dosages levels. In studies with multiple measurements of performance along several days^10^,^16^,^31^, we used the results obtained on the day after first acquisition.

Means and standard deviations (or standard errors) were obtained from each study to estimate the pooled effect sizes (Cohen’s *d*s) of the treatment effect on intact and impaired rodents. Confidence intervals for each study’s effect size, and pooled effect sizes and their confidence intervals were calculated using the common practice of weighting different studies according to their inverse variance^32^,^33^. If within a study, multiple tasks were conducted on different animals, n sizes were based on the total number of organisms taking part in the study, such that each study received weight according to the number of organisms tested. Additionally, in order to test for publication bias we plotted the effect size of each experiment by its sample size. The symmetry of such ‘funnel plots’ was assessed to see if the effect decreased with increasing sample size^34^,^35^.

To examine moderating effects we conducted Weighted Least Squares (WLS) regressions, which included the duration of *H. perforatum* administration (number of consecutive days), dosage level, type of memory predominantly assessed, and motivation (appetitive versus aversive) as predictors. In this latter examination we needed to delve into the properties of individual tasks within each study because some have used multiple task types. Therefore, each observation was further weighted according to the number of tasks within a study (the weight score was divided by the number of tasks). In light of the small number of studies, each of the four moderators (duration, dosage, memory type, and motivation) was individually entered into a separate regression model as a predictor.

## Results

**Figure 1:**
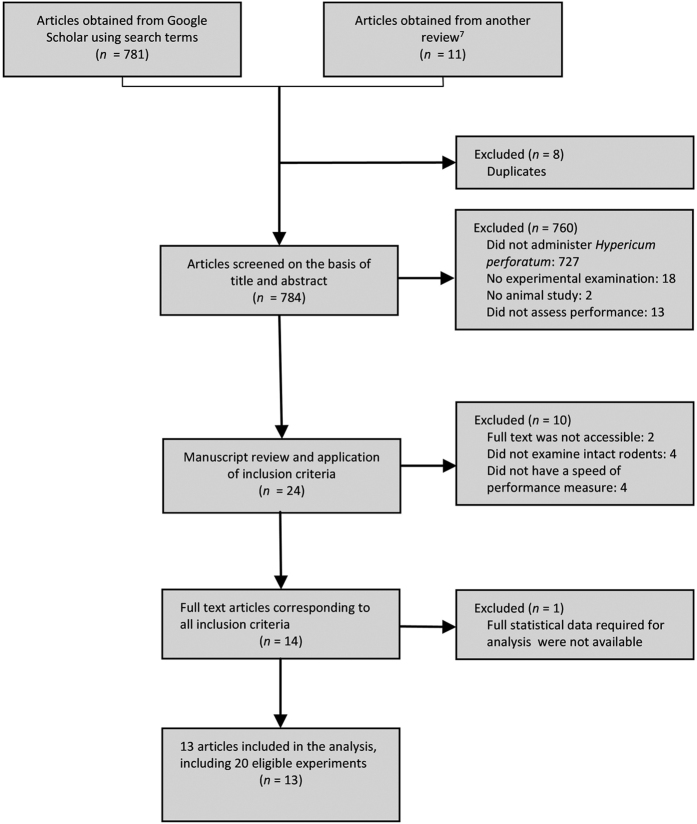
Flow diagram of literature search (based on Moher *et al.*) 57.

The literature search provided a total of 792 citations, of which 13 studies met the criteria and were included in the review. Figure 1 provides a complete flow diagram of this process. The 13 reviewed studies (each published as a separate paper between the years 2000 and 2014)^9^,^10^,^11^,^12^,^13^,^16^,^31^,^36^,^37^,^38^,^39^,^40^,^41^ involved 20 experimental comparisons. Tested animals had access to food and water and were kept in ample temperature and humidity prior to the experiment. All studies, except for two, used rats (Prakash *et al.*, 2010 38 used mice and Klusa *et al.*, 2011 9 used both rats and mice). The number of animals in each experimental condition varied between 6 and 16 (*M* = 8.3, *SD* = 2.8). No information on blinding of experimenters and data evaluators was available. Table 1 includes the detailed characteristics of each study.

Overall, for non-impaired organisms *H. perforatum* was administered to 289 healthy intact rodents (maximal dosages groups: *n* = 170) and placebo to 200 healthy intact rodents. Additionally, nine studies (involving 13 experiments) also compared the intact animals - as a control group - to a group in which cognitive performance had been impaired. Across experiments, the predominant means of impairment was by restraint-induced stress (*k* = 6); others were cortisol induced stress (*k* = 4), scopolamine, sodium nitrite, and electroconvulsive shock induced amnesia (*k* = 2), and diabetes (*k* = 1). With the exception of two studies which administrated *H. perforatum* acutely (in 1 day), others maintained daily *H. perforatum* administration sub-chronically (4 days) to chronically (from 21 to even 64 days) before measuring performance. Daily dosages varied greatly, between 1 to 350 mg/kg.

**Figure 2:**
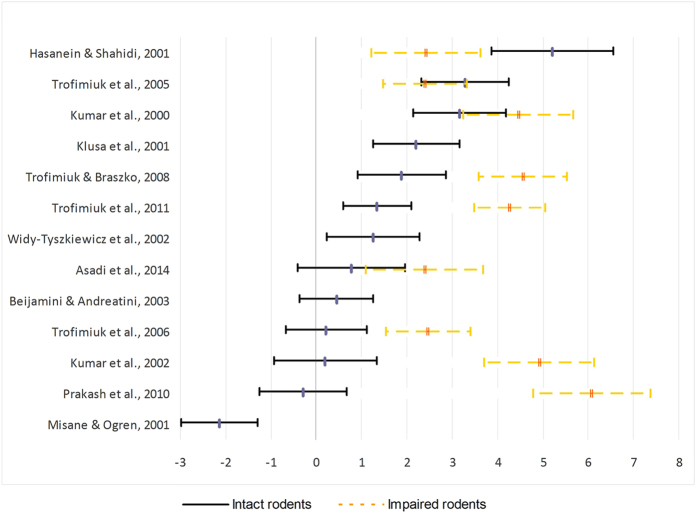
Effect sizes (Cohen’s d’s) and 95% confidence intervals for the difference between the effect of *Hypericum perforatum* and placebo on cognitive performance of intact and impaired rodents.

Figure 2 illustrates the effect size and 95% confidence interval for each of the 13 studies. Across studies, there was a large positive effect of *H. perforatum* on intact rodents’ performance (mean *d* = 1.11) which was statistically significant (95% CI: 0.84 – 1.37), indicating that administration considerably enhanced memory processes among healthy, intact, rodents. For the impaired rodents the effect was larger (mean *d* = 3.70, 95% CI: 3.35 – 4.04). This was true both for rodents whose performance was impaired by restraint stress (mean *d* = 3.10, 95% CI: 2.68 – 3.52) or by other means (mean *d* = 4.04, 95% CI: 3.62 – 4.46). The differences between the effect for intact versus impaired rodents were statistically significant (intact vs. restraint stress: *z* = 6.56, *p* < 0.001; intact vs. other means of impairment: *z* = 8.25, *p* < 0.001). Still, there was large variance in the relative influence on intact and impaired rodents. In five out of nine studies, the administration of *H. perforatum* led to significantly higher performance levels for impaired rodents; in three studies, there was no significant difference; while in one study intact rodents benefited significantly more than impaired ones.

In addition to the effect size estimation, heterogeneity was assessed using the common measurements of *Q* and *I*^2 ^42. Cochran’s *Q* test yielded significant heterogeneity for both intact (*Q* = 154, *p* < 0.001) and impaired (*Q* = 46, *p* < 0.001) rodent designs. The *I*^2^ index also showed large heterogeneity for the intact (92%) and impaired (83%) experimental comparisons. While not proving the existence of moderators driving heterogeneity, these results strongly invite further investigations of the variability across studies^43^.

As noted above, one major difference between experiments involved the type of memory assessed. The majority of the reviewed studies (*k* = 11) examined reference memory (i.e., recalling trial fixed features) and because of this the overall effect size reflects differences in reference memory to a greater extent than other types of memory. Hence, we also separately analyzed the tasks assessing each memory type. In studies of reference memory we found an effect size of *d* = 1.06 (95% CI: 0.76 – 1.35) for intact rodents, and *d* = 3.31 (95% CI: 2.88 – 3.73) for the impaired ones. In studies assessing primarily working memory there was also a large effect size for healthy intact rodents, *d* = 1.54 (95% CI: 0.95 – 2.14), and a larger effect size for impaired rodents, *d* = 4.38 (95% CI: 3.77 – 4.99) (one study focused on recognition memory with the results being similar, intact *d* = 1.75; impaired *d* = 2.75).

Weighted Least Squares (WLS) regression analyses were conducted to assess possible moderators within the intact healthy rodents. First, we compared tasks focusing on reference versus working memory (and excluding the single study examining recognition memory): the results show no significant moderating effect (*β* = 0.12, *p* = 0.62). Secondly, an examination of appetitive versus aversive motivation similarly showed no moderating effect (*β* = 0.07, *p* = 0.78). Finally, there was no moderating effect of duration (*β* = 0.26, *p* = 0.27) or dosage (*β* = 0.13, *p* = 0.59). For the impaired rodents also, neither memory type, motivation, duration, nor dosage were related to the effect size (*p’s* > 0.38).

An alternative analysis was conducted taking into consideration not only the maximal dosage, but all dosages (specifically, each dosage group was examined separately and weighted based on the number of different dosage conditions in each study, as indicated above). The effect sizes were similar to those reported above for maximal dosages, both for the intact rodents, *d* = 1.09 (95% CI: 0.83 – 1.35); and for the impaired ones, *d* = 3.58 (95% CI: 3.23 – 3.93). WLS regression analyses, analogous to those mentioned above, also did not yield any significant moderator.

**Figure 3:**
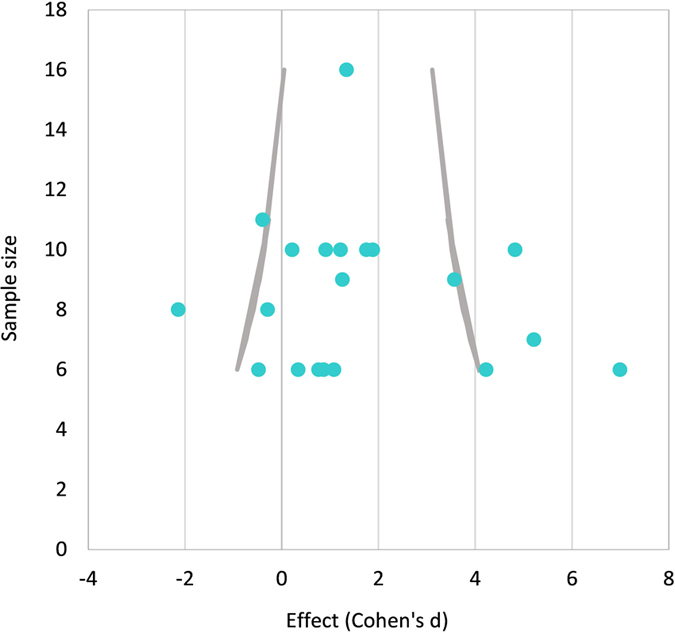
Funnel plot of experimental comparisons on intact rodents (maximal dosages only, different tasks shown separately). Margins are at *Z* = 3 (99.7%).

Finally, we tested whether the effect size is inflated due to publication bias (i.e., small sample studies with large effect sizes), using the method proposed by Egger^34^, which examines the relation between the studies effect size and their sample size. With no publication bias, studies with the largest *n* are plotted near the average effect size, and smaller studies are spread evenly on both sides, creating a roughly funnel-shaped distribution. The funnel plot, based on all tasks involving intact rodents, is shown in Fig. 3. While the funnel plot is roughly symmetric, reflecting little relation between sample size and effect size, there were four outliers with small sample sizes and extreme results (|z| > 3; 3 positive, 1 negative). Excluding these four cases, a similar size effect remains (*d* = 1.06, 95% CI: 0.78 – 1.35), emphasizing the validity of the main effect for intact rodents.

## Discussion

A meta-analytic summary of preclinical studies revealed that though primarily considered as a medication for depression, *H. perforatum* seems to have large nootropic effects. As might be expected, across studies the effect was more pronounced in rodents with impaired cognitive performance, either due to stress or amnestic agents. However, particularly for the stress-impaired rodents there was large heterogeneity between studies and only in about a third of the studies was the effect significantly higher for the impaired compared to the intact rodents. Classic antidepressant drugs, such as SSRIs and SNRIs, are not usually considered to possess nootropic traits, unless administered to depressed or anxious populations, in whom they can reduce cognitive biases and restore normal cognitive performance^44^,^45^. Our results suggest that at least for *H. perforatum*, this assumption should be re-evaluated.

Interestingly, the nootropic effect was observed in tasks involving different characteristics: first, the effect was observed both in tasks assessing reference memory as well as in tasks assessing primarily working memory. Secondly, *H. perforatum* enhanced performance both in tasks involving appetitive and aversive stimuli. This implies that the effect of *H. perforatum* on performance is not mediated solely by enhanced avoidance responses due to its effect on the mood system^37^, but rather that it modulates broader learning and memory processes.

Our view is that two basic aspects contribute to this nootropic effect for healthy intact rodents: First, there is extensive documentation that performance-related stress may impinge upon the achievements of intact organisms as well, thus reducing it may have positive effects^5^,^46^. In addition to its effect on a variety of neurotransmitters noted above, *H. perforatum* also has a long term influence on the expression of genes involved in the regulation of the hypothalamic-pituitary-adrenal (HPA) axis, which leads to reduced plasma adrenocorticotropic hormone and corticosterone level^46^ and inhibits stress-induced increases in gene transcription in the hippocampus and the pituitary^46^. An additional neuroendocrine interaction is via suppression of interleukin (IL-6) release, which inhibits substance-P mediated stress responses^47^. These diverse stress-related mechanisms are consistent with our finding that the nootropic effect was larger in stress-induced rodents. Secondly, *H. perforatum* may have additional nootropic effects that should be further investigated. In part, these processes may be specific to spatial tasks: *H. perforatum* uniquely upregulates 5-HT_2_ receptors, an effect which is thought to contribute to the enhancement of spatial memory^41^,^48^. Alternatively, it may have a general effect on memory performance, via its dopaminergic activity^27^, or due to its GABAergic traits modulating working memory^49^, or combined GABA and glutamate neuromechanisms regulating NMDA receptors^18^.

Limitations of the current study include the heterogeneity of the tasks performed, and the overall small number of studies. As well, as with any meta-analysis one should consider the likelihood that studies with null results were not published, thus inflating the effect size found. Our analysis using Egger’s method^34^ suggests marginal inflation of effect size in the present investigation, though it does not completely rule out the possibility of a drawer effect. Still, it should be stressed that in the majority of the reviewed studies the underlying assumption was an interaction effect, such that there would be a positive effect of *H. perforatum* on performance for the stress-impaired rodents and not for the intact ones. Nevertheless, a considerable and unexpected positive effect emerged for the intact control group.

In light of these findings it is interesting to consider two human studies examining the effect of *H. perforatum* administration on cognition^14^,^15^. The first study involved 12 healthy volunteers, who underwent three within-subject conditions of a single acute dose (Placebo, *H. perforatum* 900 mg or *H. perforatum* 1800 mg). No significant effect of *H. perforatum* was obtained in tasks assessing several cognitive functions (working memory, delayed recall, and reaction time). Furthermore, the highest dose (1800 mg) even impaired performance in some of the tasks^14^. The second study was also based on a small sample of 12 healthy volunteers, and examined the effect of 14 days of *H. perforatum* administration (standard antidepressant dosage containing 900 μg hypericin). No effects were found in cognitive tasks and physiological measures of autonomic nervous system activity^15^.

What seems to differentiate the former study from most of the animal studies in our review is that it had only a single administration of *H. perforatum*. For SSRIs and *H. perforatum* as well, the neurochemical effects are expected to build up and reach significance only after a couple of weeks^50^,^51^. In addition, it is worth noting that both human studies used very large dosages, about the same as those recommended for individuals suffering from moderate to severe depression^52^. Potentially, given the U shape effect found for some of the behavioral outcomes of *H. perforatum*^53^, smaller dosages may be more effective. Finally, the tasks used in the human literature markedly differed from those used in the rodent literature, being less based on learning and more on basic working memory capacity^54^,^55^. Because of the presumed attention-focusing effects of *H. perforatum*, the effects in human may be more pronounced in complex learning or interference tasks^56^. Still, despite these potential differences, the gap between the nootropic effects that we observe in rodent studies and the absence of the effect in human studies remains unclear.

## Additional Information

**How to cite this article**: Ben-Eliezer, D. and Yechiam, E. Hypericum perforatum as a cognitive enhancer in rodents: A meta-analysis. *Sci. Rep.* **6**, 35700; doi: 10.1038/srep35700 (2016).

## Supplementary Material

Supplementary Information

## Figures and Tables

**Table 1:** Preclinical studies examining the effect of *Hypericum perforatum* on cognitive performance of healthy intact rodents.

Authors, year	*H. perforatum* maximal dose and admin. Duration	Rodent age (months)	Task(s)^a^	No. of healthy animals (max dose)	Impairment method	No. of impaired animals (max dose)
Kumar *et al.*, 2000^10^	200 mg/kg × 3 days^c^	Adults	Active avoidance response, passive avoidance learning^b^	72	Drug induced amnesia	48
Klusa *et al.*, 2001^9^	50 mg/kg × 8 days^c^	Adults	Conditioned avoidance response, passive avoidance learning^b^	39	—	—
Misane & Ogren, 2001^37^	30 mg/kg × 1 day	Adults	Passive avoidance learning test^b^	16	—	—
Kumar *et al.*, 2002^16^	200 mg/kg × 3 days^c^	Adults	Active avoidance response, passive avoidance learning^b^	24	Electroconvulsive shock induced amnesia	24
Widy-Tyszkiewicz *et al.*, 2002^41^	13 mg/kg × 63 days^c^	6 m	Morris water maze^58^	17	—	—
Beijamini & Andreatini, 2003^31^	300 mg/kg × 7 days^c^	Adults	Elevated T maze^59^ ^b^	48	—	—
Trofimiuk *et al.*, 2005^39^	350 mg/kg × 21 days	2 m	Morris water maze^58^, Object recognition test^60^	40	Restraint stress; cortisol-induced stress	78
Trofimiuk *et al.*, 2006^12^	350 mg/kg × 21 days	2 m	Passive avoidance learning test^b^	20	Restraint stress; cortisol-induced stress	40
Trofimiuk & Braszko, 2008^11^	350 mg/kg × 21 days	2 m	Morris water maze^58^	20	Restraint stress; cortisol-induced stress	41
Prakash *et al.*, 2010^38^	200 mg/kg × 21 days	Adults	T maze^59^	16	Restraint stress	16
Hasenein & Shahidi, 2011^36^	25 mg/kg × 30 days^c^	3–4 m	Passive avoidance learning test^b^	14	Drug induced diabetes	14
Trofimiuk *et al.*, 2011^40^	350 mg/kg × 21 days	2 m	Barnes maze^61^	32	Restraint stress; cortisol-induced stress	64
Asadi *et al.*, 2014^13^	350 mg/kg × 7 days	Adults	Passive avoidance learning test^62^^ ^b	12	Restraint stress	12

Note: C – Control group (Intact rodents), H – *H. perforatum* group (Intact rodents), I –Impaired group.

IH – Impaired and *H. perforatum* group.

^a^Mean latency or duration of performance was measured, unless otherwise noted.

^b^Duration of avoiding a dark compartment where rats had previously undergone electric shock. Effect size was inversed.

^c^Multiple dosages of *H. perforatum* were administrated (maximal dose is noted).
