# Supplementary material for: Hypericum perforatum as a cognitive enhancer in rodents: A meta-analysis
Source: Sci Rep. 2016 Oct 20;6:35700. doi: 10.1038/srep35700 (PMC5071825; doi:10.1038/srep35700)
Supplement: Supplementary Information [file srep35700-s1.doc]

**Supplementary Materials**

**Hypericum perforatum as a cognitive enhancer in rodents: A meta-analysis**

Daniel Ben-Eliezer^1^, Eldad Yechiam*^1^

^1^ Technion – Israel Institute of Technology, Haifa

* Corresponding author: Eldad Yechiam, Max Wertheimer Minerva Center for Cognitive Studies, Faculty of Industrial Engineering and Management, Technion - Israel Institute of Technology, Haifa 3200003, Israel. Phone: (972) 4-829-4420, Fax: (972) 4-829-5688,
Email: yeldad@tx.technion.ac.il

Supplementary table S1: Mapping of tasks into predominant memory requirements (reference versus working memory) and motivation (appetitive versus aversive stimulus). Studies are ordered according to the effect size in Table 1.

| First author, year | Task(s)^a^ | Memory | Motivation |
| --- | --- | --- | --- |

| Kumar et al., 2000 | Active avoidance response | Reference | Aversive |
| --- | --- | --- | --- |
|  | Passive avoidance learning | Reference | Aversive |
| Klusa et al., 2001 | Conditioned avoidance response | Reference | Aversive |
|  | Passive avoidance learning | Reference | Aversive |
| Misane & Ogren, 2001 | Passive avoidance learning | Reference | Aversive |
| Kumar et al., 2002 | Active avoidance learning | Reference | Aversive |
|  | Passive avoidance response | Reference | Aversive |
| Widy-Tyszkiewicz  et al., 2002 | Morris water maze^a^ | Reference | Aversive |

| Beijamini & Andreatini, 2003 | Elevated T maze | Reference | Aversive |
| --- | --- | --- | --- |
| Trofimiuk, et al. 2005 | Morris water maze | Reference | Aversive |
|  | Object recognition test | Recognition | Appetitive |

| Trofimiuk et al., 2006 | Passive avoidance learning | Reference | Aversive |
| --- | --- | --- | --- |

| Trofimiuk & Braszko, 2008 | Morris water maze^a^ | Working memory | Aversive |
| --- | --- | --- | --- |
| Prakash et al., 2010 | T maze | Reference | Appetitive |

| Hasenein & Shahidi, 2011 | Passive avoidance learning | Reference | Aversive |
| --- | --- | --- | --- |

Note: ^a^ Morris Water Maze assesses reference memory, but may also assess working

| Trofimiuk et al., 2011 | Barnes maze | Working memory | Aversive |
| --- | --- | --- | --- |
| Asadi et al., 2014 | Passive avoidance learning | Reference | Aversive |
|  |  | | |

memory, depending on the version.
